# Using multi-tissue transcriptome-wide association study to identify candidate susceptibility genes for respiratory infectious diseases

**DOI:** 10.3389/fgene.2023.1164274

**Published:** 2023-03-20

**Authors:** Xiaobo Zhu, Yixin Zou, Linna Jia, Xiangyu Ye, Yanzheng Zou, Junlan Tu, Juntong Li, Rongbin Yu, Sheng Yang, Peng Huang

**Affiliations:** ^1^ The People’s Hospital of Danyang, Affiliated Danyang Hospital of Nantong University, Zhenjiang, China; ^2^ Department of Epidemiology, Center for Global Health, School of Public Health, Nanjing Medical University, Nanjing, China; ^3^ Department of Biostatistics, Center for Global Health, School of Public Health, Nanjing Medical University, Nanjing, China

**Keywords:** transcriptome-wide association study, IAV, measles, mumps, rubella, multiple tissues, genome-wide association studies, gene expression

## Abstract

**Objective:** We explore the candidate susceptibility genes for influenza A virus (IAV), measles, rubella, and mumps and their underlying biological mechanisms.

**Methods:** We downloaded the genome-wide association study summary data of four virus-specific immunoglobulin G (IgG) level data sets (anti-IAV IgG, anti-measles IgG, anti-rubella IgG, and anti-mumps virus IgG levels) and integrated them with reference models of three potential tissues from the Genotype-Tissue Expression (GTEx) project, namely, whole blood, lung, and transformed fibroblast cells, to identify genes whose expression is predicted to be associated with IAV, measles, mumps, and rubella.

**Results:** We identified 19 significant genes (ULK4, AC010132.11, SURF1, NIPAL2, TRAP1, TAF1C, AC000078.5, RP4-639F20.1, RMDN2, ATP1B3, SRSF12, RP11-477D19.2, TFB1M, XXyac-YX65C7_A.2, TAF1C, PCGF2, and BNIP1) associated with IAV at a Bonferroni-corrected threshold of *p* < 0.05; 14 significant genes (SOAT1, COLGALT2, AC021860.1, HCG11, METTL21B, MRPL10, GSTM4, PAQR6, RP11-617D20.1, SNX8, METTL21B, ANKRD27, CBWD2, and TSFM) associated with measles at a Bonferroni-corrected threshold of *p* < 0.05; 15 significant genes (MTOR, LAMC1, TRIM38, U91328.21, POLR2J, SCRN2, Smpd4, UBN1, CNTROB, SCRN2, HOXB-AS1, SLC14A1, AC007566.10, AC093668.2, and CPD) associated with mumps at a Bonferroni-corrected threshold of *p* < 0.05; and 13 significant genes (JAGN1, RRP12, RP11-452K12.7, CASP7, AP3S2, IL17RC, FAM86HP, AMACR, RRP12, PPP2R1B, C11orf1, DLAT, and TMEM117) associated with rubella at a Bonferroni-corrected threshold of *p* < 0.05.

**Conclusions:** We have identified several candidate genes for IAV, measles, mumps, and rubella in multiple tissues. Our research may further our understanding of the pathogenesis of infectious respiratory diseases.

## Introduction

Although respiratory infections are largely preventable causes of illness and death, they remain the leading cause of death from infectious diseases worldwide, ranking fifth among all total causes of death (GBD 2015 LRI Collaborators, 2017). Influenza A virus (IAV), measles virus, rubella virus, and mumps virus infect a wide range of hosts, are highly transmissible and are prone to a mutation that can cause pandemics in a short period of time, placing an enormous burden and pressure on public health systems (Marshall and Plotkin, 2019; Harrington et al., 2021; Iacobucci, 2022; Raghunathan and Orenstein, 2022). The incidence of IAV, measles, mumps, and rubella has decreased drastically following the implementation of vaccination programs. However, the segmented genome of IAV allows for easy recombination and antigenic shift, resulting in novel antigens (Bouvier and Palese, 2008). Thus, the ability of IAV to rapidly evolve can result in highly pathogenic viral strains. Measles and rubella remain endemic in many countries, leading to the importation of cases and occasional local transmission within China, and the resurgence of mumps has recently been reported, with outbreaks still occurring and challenges remaining while controlling these diseases (Kauffmann et al., 2021). There is considerable variation in the severity of respiratory infections caused by viral infections. There are major determinants of this variability, such as intrinsic virus pathogenicity, acquired host factors (e.g., immunity and coexisting conditions), and innate host susceptibility. While viral genetic determinants of respiratory disease severity and host immunity have been well studied, host genetic determinants have been much less explored (Kennedy et al., 2014; Voigt et al., 2018; Sabikunnahar et al., 2022).

Genome-wide association studies (GWAS) are effective methods for understanding the genetic basis of many complex traits in common human diseases (Hayes, 2013). In particular, these have proven to be well-suited for identifying common single-nucleotide polymorphism (SNP) variants with moderate to large effects on phenotypes (Gorlova et al., 2022). However, the specific biological mechanisms and functional consequences of many of the genetic variants identified by the GWAS remain unclear, especially their role in disease severity; that is, GWAS methods may miss small effect–trait associations. Gene expression is an intermediate phenotype between genetic variation and an underlying disease predisposition trait (Albert and Kruglyak, 2015). Many genetic variants affect complex traits by regulating gene expressions. Unfortunately, large-scale expression–trait associations are hampered by sample availability and cost, as well as intrinsic factors and small effects. Therefore, to address these issues, transcriptome-wide association studies (TWASs) were developed, which integrate gene expression into large-scale GWAS (Derks and Gamazon, 2020). Through extensive simulation of existing GWAS data, TWASs have identified candidate genes associated with mental disorders (Thériault et al., 2018), calcified aortic stenosis (Liu et al., 2020), pancreatic cancer (Lin et al., 2017), and inflammatory biological age (Zhang et al., 2022). However, for many complex traits, biologically relevant tissues are unknown. Most existing studies identify gene–trait associations on the basis of a single tissue, whereby the significant gene effects that are identified get inflated. In addition, it has been shown that eQTLs with larger effects tend to regulate gene expressions in multiple tissues. The TWAS analysis of multiple tissues improves the accuracy of the results.

There have been some studies exploring the association of respiratory infectious diseases with some human tissues. Pulmonary inflammation and airway epithelial injury are hallmarks of human pulmonary infectious diseases. The association of HLA alleles with respiratory infectious diseases in lung cells has been confirmed by Zhang et al. (2022). Moreover, Xiao et al. (2023) found that interleukin-11 (IL-11) as a fibrotic factor may be associated with respiratory diseases leading to respiratory failure or even death. These potential connections deserve our attention.

In this study, we used TWASs to explore genes associated with IAV, measles, rubella, and mumps. Given that respiratory infectious diseases are associated with multiple tissues, we used eQTL reference panels for three tissues, namely, whole blood, lung, and transformed fibroblast cells, and pooled the GWAS data for IAV, measles, rubella, and mumps to identify tissue-specific susceptibility genes. This study lays the foundation for further understanding the pathogenesis of these four viruses.

## Methods

### GWAS data collection and processing

**FIGURE 1:**
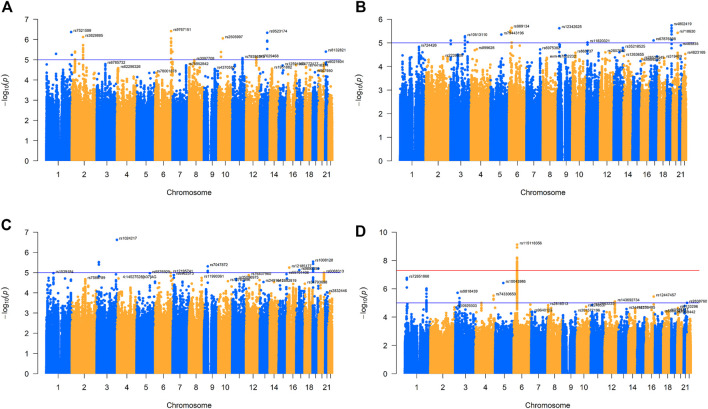
Manhattan plot for four GWAS of respiratory infectious diseases. **(A)**. Manhattan plot of IAV. **(B)**. Manhattan plot of measles. **(C)**. Manhattan plot of mumps. **(D)**. Manhattan plot of rubella.

We collected four virus-specific immunoglobulin G (IgG) level data sets (anti-IAV IgG, anti-measles IgG, anti-rubella IgG, and anti-mumps virus IgG levels) from the NHGRI-EBI GWAS directory (https://www.ebi.ac.uk/gwas/downloads/summary-statistics). These data sets were originally obtained from 1,000 healthy individuals (MI), and their serum prevalence rates were 77.7%, 88.5%, 93.5%, and 91.2%, respectively, for these four viruses (Scepanovic et al., 2018). Considering that the main unit of anti-IAV IgG quantitative unit is S/CO (signal/cut-off ratio), the main unit of anti-measles and mumps virus IgG level is IA (index antibody), and the main unit of anti-rubella virus IgG is UI/mL. We also use SD as the change unit. Then, we screened the above data for single-nucleotide polymorphisms (SNPs) for further analysis. We retained the following SNPs: 1) autosomes (1–22), 2) minimum allele frequencies (MAF) greater than 0.001, and 3) more than 70% of observers having these specific SNPs (Yang and Zhou, 2020; Yang and Zhou, 2022). We also plotted Manhattan plots and q-q plots for the processed GWAS data sets for four traits to show the frequency and distribution of their genes (Figure 1; Supplementary Figure S1).

### Transcriptome-wide association analysis

We used FUSION to perform relevant TWAS tissue analysis using GWAS summary data formatted after the processing of whole blood, lung, and transformed fibroblast cells (Gusev et al., 2016). Specifically, FUSION uses the pre-calculated gene expression weight to perform summary statistics with respiratory infectious diseases GWAS and calculates the association between each gene and respiratory infectious diseases. Association statistics are defined as TWAS *z*-scores and estimated as follows:

$$Z_{TWAS}={WZ/\left(W\sum_{S,S}W^{t}\right)}^{1/2}.$$

The weight of Z and W plays a crucial role in the distribution of Z_TWAS_. In the formula, Z is the estimated value of the SNPs for the disease under study, w^t^ is the weight, and s is the linkage disequilibrium between all SNPs. It is worth noting that Z_TWAS_ is only well calibrated under the zero model of Z ∼ N (0, Σs,s) (mean 0, unit variance 0). The reference panel that we used came from the 1000 Genomes Project in Europe (Speed et al., 2020). We also downloaded from FUSION (http://gusevlab.org/projects/fusion/) pre-computed gene expression weights based on GTEx v7 feature weights and used these for the research of each tissue. Based on the highest cross-validation *R* ^2^, FUSION selects the best expression prediction model among the top eQTL, the best linear predictor (BLUP) (Gusev et al., 2016), the Bayesian linear mixed model (BSLMM) (Zhou et al., 2013), the least absolute shrinkage and selection operator (LASSO) (Tibshirani, 1996; Veturi and Ritchie, 2018), and the elastic net (Hui and Hastie, 2005) to calculate the SNP expression weight within a 1 Mb window for a given gene. We also applied the Bonferroni correction to explain multiple hypotheses and set the *p*-value threshold to 0.01.

## Results

### Multi-tissue transcriptome-wide significant genes of IAV

**FIGURE 2:**
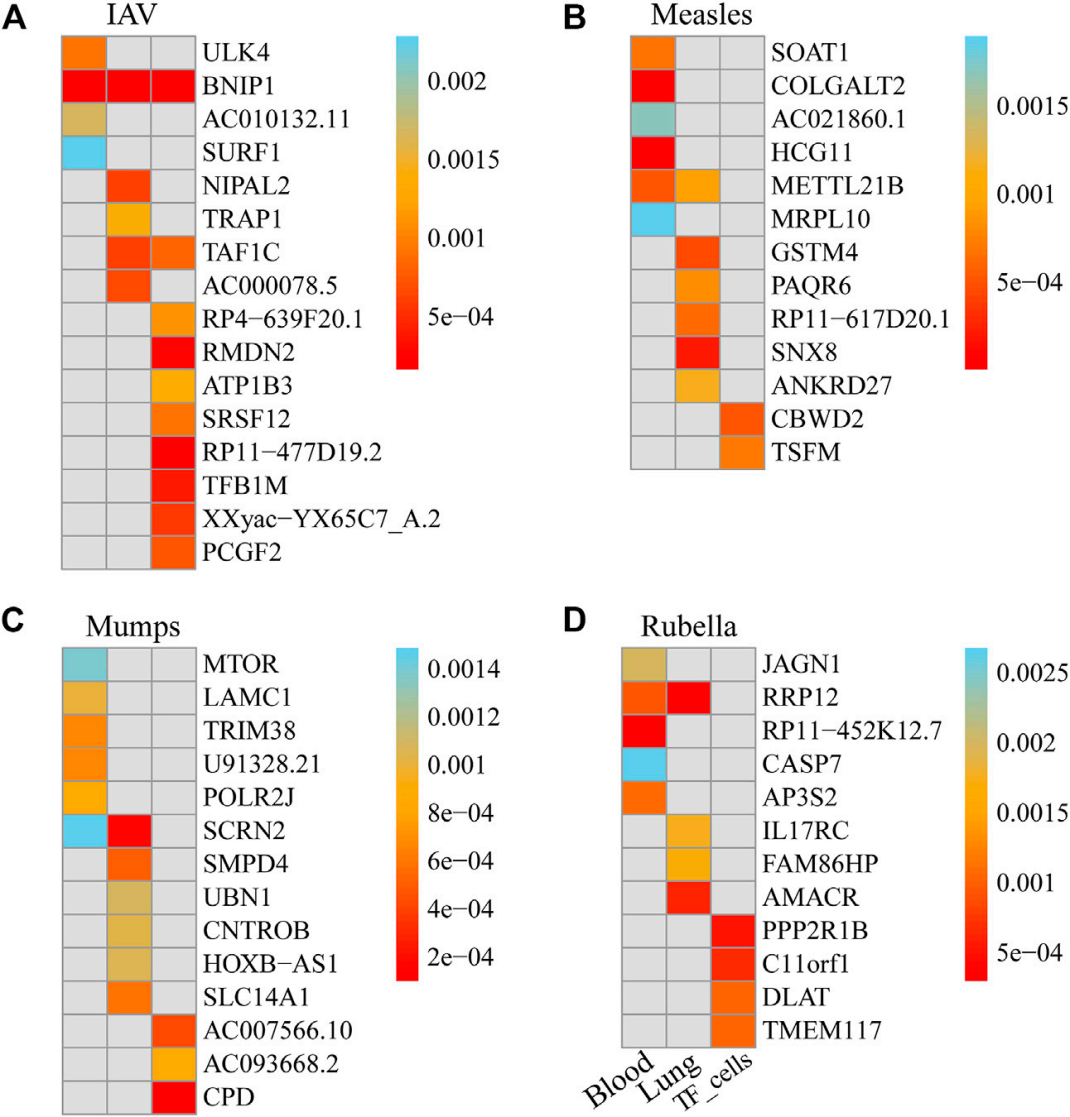
Genes significantly associated with the risk of four respiratory infectious diseases in each tissue. **(A)**. Genes significantly associated with the risk of IAV in each tissue. **(B)**. Genes significantly associated with the risk of measles in each tissue. **(C)**. Genes significantly associated with the risk of mumps in each tissue. **(D)**. Genes significantly associated with the risk of rubella in each tissue.

We used FUSION to assess the relationship between the predictive gene expression and IAV. After Bonferroni correction, we found that 19 genes in the 3 tissues were significantly associated with IAV (Figure 2A; Table 1). Specifically, in the whole blood, we found ULK4 (*p*_adjusted_ = 0.012), AC010132.11 (*p*_adjusted_ = 0.022), and SURF1 (*p*_adjusted_ = 0.030) to be IAV-related genes, while in the lung tissue, we found NIPAL2 (*p*_adjusted_ = 0.019), TRAP1 (*p*_adjusted_ = 0.042), TAF1C (*p*_adjusted_ = 0.018), and AC000078.5 (*p*_adjusted_ = 0.020) to be significantly associated with IAV. Other significant genes included RP4-639F20.1 (*p*_adjusted_ = 0.041), RMDN2 (*p*_adjusted_ = 0.009), ATP1B3 (*p*_adjusted_ = 0.050), SRSF12 (*p*_adjusted_ = 0.033), RP11-477D19.2 (*p*_adjusted_ = 0.007), TFB1M (*p*_adjusted_ = 0.015), XXyac-YX65C7_A.2 (*p*_adjusted_ = 0.021), TAF1C (*p*_adjusted_ = 0.030), and PCGF2 (*p*_adjusted_ = 0.027) in the transformed fibroblast cells. However, we found that BNIP1 expressed in the whole blood, lung tissue, and transformed fibroblast cells was significantly associated with IAV.

**TABLE 1:** Genes significantly associated with the risk of IAV.

Tissue	Gene	CHR	MODEL	TWAS.Z	TWAS.P	*P-Bonferroni*
Blood	ULK4	3	ENet	−3.319	9.050E-04	0.012
Blood	BNIP1	5	Top1	−3.763	1.680E-04	0.002
Blood	AC010132.11	7	Top1	3.142	1.680E-03	0.022
Blood	SURF1	9	LASSO	3.050	2.290E-03	0.030
Lung	BNIP1	5	LASSO	−3.730	1.910E-04	0.006
Lung	NIPAL2	8	LASSO	3.422	6.220E-04	0.019
Lung	TRAP1	16	ENet	−3.192	1.411E-03	0.042
Lung	TAF1C	16	ENet	3.431	6.010E-04	0.018
Lung	AC000078.5	22	ENet	−3.409	6.520E-04	0.020
T.F cells	RP4-639F20.1	1	LASSO	−3.252	1.140E-03	0.041
T.F cells	RMDN2	2	ENet	3.658	2.540E-04	0.009
T.F cells	ATP1B3	3	Top1	3.199	1.380E-03	0.050
T.F cells	BNIP1	5	ENet	−3.714	2.040E-04	0.007
T.F cells	SRSF12	6	Top1	3.314	9.200E-04	0.033
T.F cells	RP11-477D19.2	6	Top1	−3.731	1.910E-04	0.007
T.F cells	TFB1M	6	ENet	−3.536	4.070E-04	0.015
T.F cells	XXyac-YX65C7_A.2	6	ENet	3.443	5.760E-04	0.021
T.F cells	TAF1C	16	ENet	3.342	8.310E-04	0.030
T.F cells	PCGF2	17	LASSO	3.367	7.600E-04	0.027

CHR, the chromosome on which the identified gene is located; MODEL, models used for imputation in FUSION; TWAS.P, *p*-values for TWAS analysis in each tissue; P-Bonferroni, *p*-values corrected by Bonferroni for TWAS analysis in each tissue.

### Multi-tissue transcriptome-wide significant genes of measles

We used FUSION to assess the relationship between predictive gene expression and measles. After Bonferroni correction, we found that 14 genes in the three tissues were significantly associated with measles (Figure 2B; Table 2). Specifically, in the whole blood, we found SOAT1 (*p*_adjusted_ = 0.018), COLGALT2 (*p*_adjusted_ = 0.000), AC021860.1 (*p*_adjusted_ = 0.044), HCG11 (*p*_adjusted_ = 0.000), METTL21B (*p*_adjusted_ = 0.014), and MRPL10 (*p*_adjusted_ = 0.049) as measles-related genes, while in the lung tissue, we found GSTM4 (*p*_adjusted_ = 0.017), PAQR6 (*p*_adjusted_ = 0.031), RP11-617D20.1 (*p*_adjusted_ = 0.024), SNX8 (*p*_adjusted_ = 0.009), METTL21B (*p*_adjusted_ = 0.035), and ANKRD27 (*p*_adjusted_ = 0.044) to be significantly associated with measles. Other significant genes included CBWD2 (*p*_adjusted_ = 0.029) and TSFM (*p*_adjusted_ = 0.041) in transformed fibroblast cells.

**TABLE 2:** Genes significantly associated with the risk of measles.

Tissue	Gene	CHR	MODEL	TWAS.Z	TWAS.P	*P-Bonferroni*
Blood	SOAT1	1	Top1	3.399	6.760E-04	0.018
Blood	COLGALT2	1	Top1	4.308	1.650E-05	0.000
Blood	AC021860.1	4	LASSO	3.137	1.710E-03	0.044
Blood	HCG11	6	LASSO	−4.459	8.220E-06	0.000
Blood	METTL21B	12	Top1	3.465	5.300E-04	0.014
Blood	MRPL10	17	ENet	−3.106	1.900E-03	0.049
Lung	GSTM4	1	ENet	−3.512	4.440E-04	0.017
Lung	PAQR6	1	ENet	3.345	8.240E-04	0.031
Lung	RP11-617D20.1	4	ENet	3.415	6.380E-04	0.024
Lung	SNX8	7	LASSO	−3.680	2.330E-04	0.009
Lung	METTL21B	12	ENet	3.314	9.210E-04	0.035
Lung	ANKRD27	19	ENet	3.252	1.150E-03	0.044
T.F cells	CBWD2	2	LASSO	3.475	5.110E-04	0.029
T.F cells	TSFM	12	ENet	−3.383	7.160E-04	0.041

CHR, the chromosome on which the identified gene is located; MODEL, models used for imputation in FUSION; TWAS.P, *p*-values for TWAS analysis in each tissue; P-Bonferroni, *p*-values corrected by Bonferroni for TWAS analysis in each tissue.

### Multi-tissue transcriptome-wide significant genes of mumps

We used FUSION to assess the relationship between predictive gene expression and mumps. After Bonferroni correction, we found that 15 genes in the three tissues were significantly associated with mumps (Figure 2C; Table 3). In mumps, we found six significant genes, namely, MTOR (*p*_adjusted_ = 0.029), LAMC1 (*p*_adjusted_ = 0.021), TRIM38 (*p*_adjusted_ = 0.014), U91328.21 (*p*_adjusted_ = 0.014), POLR2J (*p*_adjusted_ = 0.018), and SCRN2 (*p*_adjusted_ = 0.031) in the whole blood; similarly, in the lung tissue, we found Smpd4 (*p*_adjusted_ = 0.022), UBN1 (*p*_adjusted_ = 0.049), CNTROB (*p*_adjusted_ = 0.046), SCRN2 (*p*_adjusted_ = 0.008), HOXB-AS1 (*p*_adjusted_ = 0.048), and SLC14A1 (*p*_adjusted_ = 0.027) as mumps-related genes, while in the transformed fibroblast cells, we found AC007566.10 (*p*_adjusted_ = 0.023), AC093668.2 (*p*_adjusted_ = 0.047), and CPD (*p*_adjusted_ = 0.005) as significantly associated with mumps.

**TABLE 3:** Genes significantly associated with the risk of mumps.

Tissue	Gene	CHR	MODEL	TWAS.Z	TWAS.P	*P-Bonferroni*
Blood	MTOR	1	ENet	−3.195	1.400E-03	0.029
Blood	LAMC1	1	Top1	−3.289	1.010E-03	0.021
Blood	TRIM38	6	Top1	−3.401	6.710E-04	0.014
Blood	U91328.21	6	Top1	−3.401	6.710E-04	0.014
Blood	POLR2J	7	Top1	−3.327	8.780E-04	0.018
Blood	SCRN2	17	ENet	3.177	1.490E-03	0.031
Lung	Smpd4	2	LASSO	3.483	4.960E-04	0.022
Lung	UBN1	16	ENet	3.266	1.090E-03	0.049
Lung	CNTROB	17	ENet	−3.285	1.020E-03	0.046
Lung	SCRN2	17	LASSO	3.738	1.850E-04	0.008
Lung	HOXB-AS1	17	LASSO	3.274	1.060E-03	0.048
Lung	SLC14A1	18	LASSO	3.436	5.910E-04	0.027
T.F cells	AC007566.10	7	Top1	−3.524	4.250E-04	0.023
T.F cells	AC093668.2	7	Top1	3.327	8.780E-04	0.047
T.F cells	CPD	17	LASSO	−3.886	1.020E-04	0.005

CHR, the chromosome on which the identified gene is located; MODEL, models used for imputation in FUSION; TWAS.P, *p*-values for TWAS analysis in each tissue; P-Bonferroni, *p*-values corrected by Bonferroni for TWAS analysis in each tissue.

### Multi-tissue transcriptome-wide significant genes of rubella

We used FUSION to assess the relationship between predictive gene expression and rubella. After Bonferroni correction, we found that 13 genes in the three tissues were significantly associated with rubella (Figure 2D; Table 4). In rubella, we found five significant genes, namely, JAGN1 (*p*_adjusted_ = 0.036), RRP12 (*p*_adjusted_ = 0.017), RP11-452K12.7 (*p*_adjusted_ = 0.006), CASP7 (*p*_adjusted_ = 0.048), and AP3S2 (*p*_adjusted_ = 0.020) in the whole blood, while in the lung tissue, we found IL17RC (*p*_adjusted_ = 0.035), FAM86HP (*p*_adjusted_ = 0.034), AMACR (*p*_adjusted_ = 0.012), and RRP12 (*p*_adjusted_ = 0.006) to be significantly associated with rubella. Similarly, in transformed fibroblast cells, we found PPP2R1B (*p*_adjusted_ = 0.022), C11orf1 (*p*_adjusted_ = 0.029), DLAT (*p*_adjusted_ = 0.045), and TMEM117 (*p*_adjusted_ = 0.046) as rubella-related genes.

**TABLE 4:** Genes significantly associated with the risk of rubella.

Tissue	Gene	CHR	MODEL	TWAS.Z	TWAS.P	*P-Bonferroni*
Blood	JAGN1	3	Top1	−3.092	1.990E-03	0.036
Blood	RRP12	10	LASSO	−3.303	9.560E-04	0.017
Blood	RP11-452K12.7	10	LASSO	−3.608	3.090E-04	0.006
Blood	CASP7	10	Top1	3.002	2.682E-03	0.048
Blood	AP3S2	15	LASSO	3.263	1.100E-03	0.020
Lung	IL17RC	3	ENet	3.126	1.770E-03	0.035
Lung	FAM86HP	3	ENet	−3.136	1.710E-03	0.034
Lung	AMACR	5	ENet	3.425	6.160E-04	0.012
Lung	RRP12	10	Top1	−3.614	3.020E-04	0.006
T.F cells	PPP2R1B	11	ENet	−3.485	4.930E-04	0.022
T.F cells	C11orf1	11	ENet	3.408	6.560E-04	0.029
T.F cells	DLAT	11	Top1	−3.282	1.031E-03	0.045
T.F cells	TMEM117	12	ENet	3.277	1.050E-03	0.046

CHR, the chromosome on which the identified gene is located; MODEL, models used for imputation in FUSION; TWAS.P, *p*-values for TWAS analysis in each tissue; P-Bonferroni, *p*-values corrected by Bonferroni for TWAS analysis in each tissue.

## Discussion

In this study, we used tissue-specific TWAS to explore the association of genetically predicted gene expression with IAV, measles, rubella, and mumps and identified 19, 14, 15, and 13 genes in three tissues as potential IAV-, measles-, mumps-, and rubella- related genes, respectively. In IAV, including three in whole blood (ULK4, AC010132.11, and SURF1), four in the lung tissue (NIPAL2, TRAP1, TAF1C, and AC000078.5), nine in the transformed fibroblast cells (RP4-639F20.1, RMDN2, ATP1B3, SRSF12, RP11-477D19.2, TFB1M, XXyac-YX65C7_A.2, TAF1C, and PCGF2), and one expressed in three tissues as significantly associated with IAV (BNIP1). In measles, including six in whole blood (SOAT1, COLGALT2, AC021860.1, HCG11, METTL21B, and MRPL10), six in the lung tissue (GSTM4, PAQR6, RP11-617D20.1, SNX8, METTL21B, and ANKRD27), and two in the transformed fibroblast cells (CBWD2, and TSFM). In mumps, including six in whole blood (MTOR, LAMC1, TRIM38, U91328.21, POLR2J, and SCRN2), six in the lung tissue (Smpd4, UBN1, CNTROB, SCRN2, HOXB-AS1, and SLC14A1), and three in the transformed fibroblast cells (AC007566.10, AC093668.2, and CPD). In rubella, including five in whole blood (JAGN1, RRP12, RP11-452K12.7, CASP7, and AP3S2), four in the lung tissue (IL17RC, FAM86HP, AMACR, and RRP12), and four in the transformed fibroblast cells (PPP2R1B, C11orf1, DLAT, and TMEM117). The above 59 genes have never been clearly reported to be associated with IAV, measles, mumps, and rubella.

TNF receptor-associated protein-1 (TRAP1), a member of the mitochondria-specific Hsp90 family, is located in the mitochondrial matrix, mitochondrial endometrium, and the intermembrane space (Cechetto and Gupta, 2000; Pridgeon et al., 2007). However, among the few real TRAP1 clients described, two are subunits of the electron transport chain complex (ETC), a component of the complex II succinate dehydrogenase subunit A/B (SDHA/B) (Sanchez-Martin et al., 2020; Sanchez-Martin et al., 2021), and complex IV cytochromes c oxidase subunit 2 (COXII) (Xiang et al., 2016; Xiang et al., 2019). The II/SDH complex is a protein complex containing iron and sulfur groups whose function is to transfer electrons from succinate to the coenzyme Q10-ubiquinone (III complex) (Bezawork-Geleta et al., 2017). TRAP1 leaves the SDH in a partially unfolded state. Inhibition of TRAP1 can release active SDH and increase its activity (Agarwal et al., 2019; Hu et al., 2020). In addition, SDH activity (Masgras et al., 2017; Hu et al., 2020) and oxygen consumption (Masgras et al., 2017) were negatively correlated with TRAP1 expression, suggesting that TRAP1 promotes the Warburg effect (Guzzo et al., 2014). It should be noted that SDH also oxidizes succinic acid to fumarate, thus integrating the TCA cycle, suggesting a broad impact of TRAP1 on mitochondrial metabolism (Guzzo et al., 2014; Hsu et al., 2016). It can be assumed that the abnormal expression of the TRAP1 gene may contribute to the development of IAV by affecting mitochondrial metabolic processes.

GSTM4 belongs to the mu class of glutathione S-transferase (GST). Compared to other GSTMs (Comstock et al., 1993), GSTM4 exhibits greater consistency in amino acid sequence but has significant differences in physicochemical properties and tissue distribution. GSTM4 does not exhibit activity comparable to standard TPS substrates, and its specific substrate has not been identified. It is well known that the GST gene is highly polymorphic to adapt to the growing number of exotic compounds (Hayes and Strange, 2000). This polymorphism can alter individual susceptibility to the disease and response to therapeutic agents. For example, the T2517C polymorphism in GSTM4 has been shown to be associated with an increased risk of lung cancer (Liloglou et al., 2002). The mechanism underlying this association is not yet clear. Currently, there are few reports of the relationship between the GSTM4 gene and the disease. Efforts may be needed to verify its role in infectious respiratory diseases.

In addition to the aforementioned genes, we have identified other candidate genes (SOAT1, HCG11, PAQR6, and AP3S2) in different tissues, of which AP3S2 has been reported to be associated with the development of type 2 diabetes (Kazakova et al., 2017). Other genes have also been implicated in cancer. For example, SOAT1 has been linked to the development of stomach cancer (Zhu et al., 2021), and pancreatic cancer (Oni et al., 2020); HCG11 may influence the development of nasopharyngeal carcinoma (Zheng et al., 2022); the PAQR6 gene has also been associated with several cancers (Yang et al., 2021). Although there is little research on these genes and respiratory infectious diseases, the potential links between these genes deserve further attention in future studies.

In summary, our results list some genes for respiratory infections that have not been studied before. Based on the previous knowledge of these genes, this means that they may be involved in host infection through transcriptional processes of susceptible genes and RNA degradation processes. This study is helpful to deepen the understanding of the pathogenesis of respiratory infectious diseases.

## Data Availability

Publicly available data sets were analyzed in this study. These data can be found here: https://www.ebi.ac.uk/gwas/downloads/summary-statistics.
