# Supplementary figures and images for: Using multi-tissue transcriptome-wide association study to identify candidate susceptibility genes for respiratory infectious diseases
Source: Front Genet. 2023 Mar 20;14:1164274. doi: 10.3389/fgene.2023.1164274 (PMC10067569; doi:10.3389/fgene.2023.1164274)

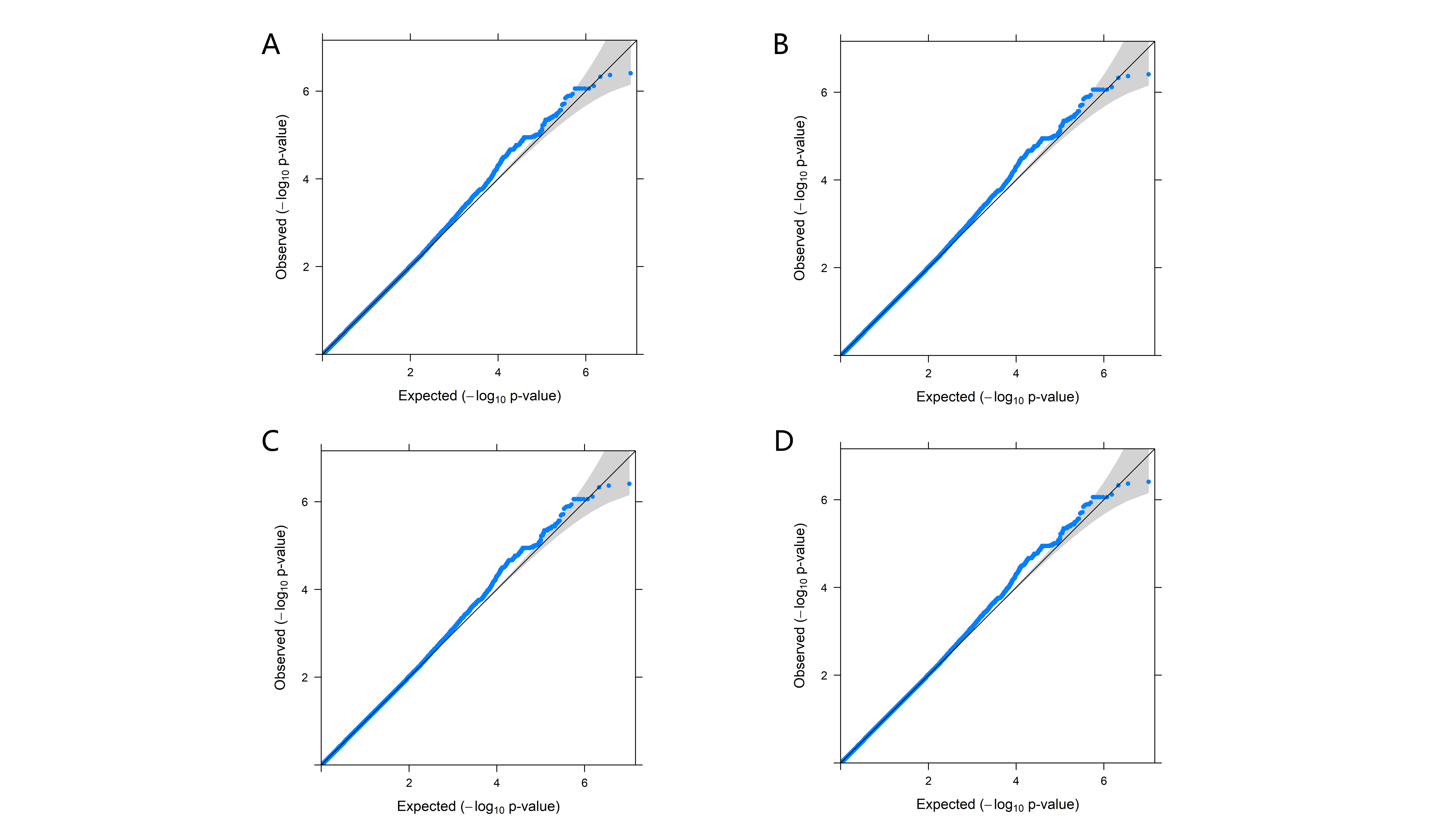

Supplement: Supplementary file 1 [file Image1.TIF]
